# Supplementary figures and images for: Predicting input signals of transcription factors in Escherichia coli
Source: Mol Syst Biol. 2025 Jul 16;21(10):1371–87. doi: 10.1038/s44320-025-00132-2 (PMC12494820; doi:10.1038/s44320-025-00132-2)

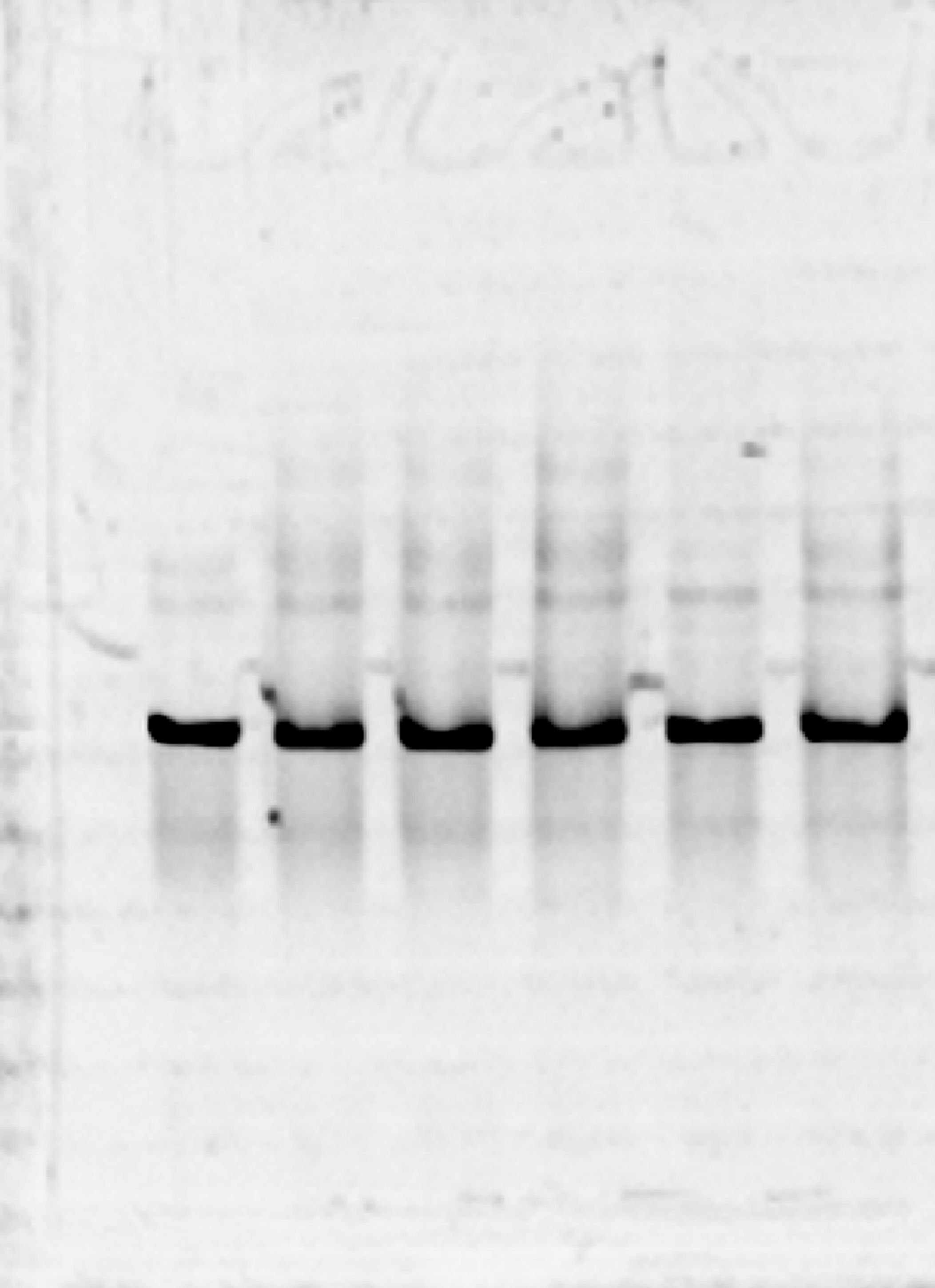

Supplement: Supplementary file 15 — Source data Fig. 6 [file 44320_2025_132_MOESM15_ESM.zip › Figure 6/Figure6B_gel1.png]

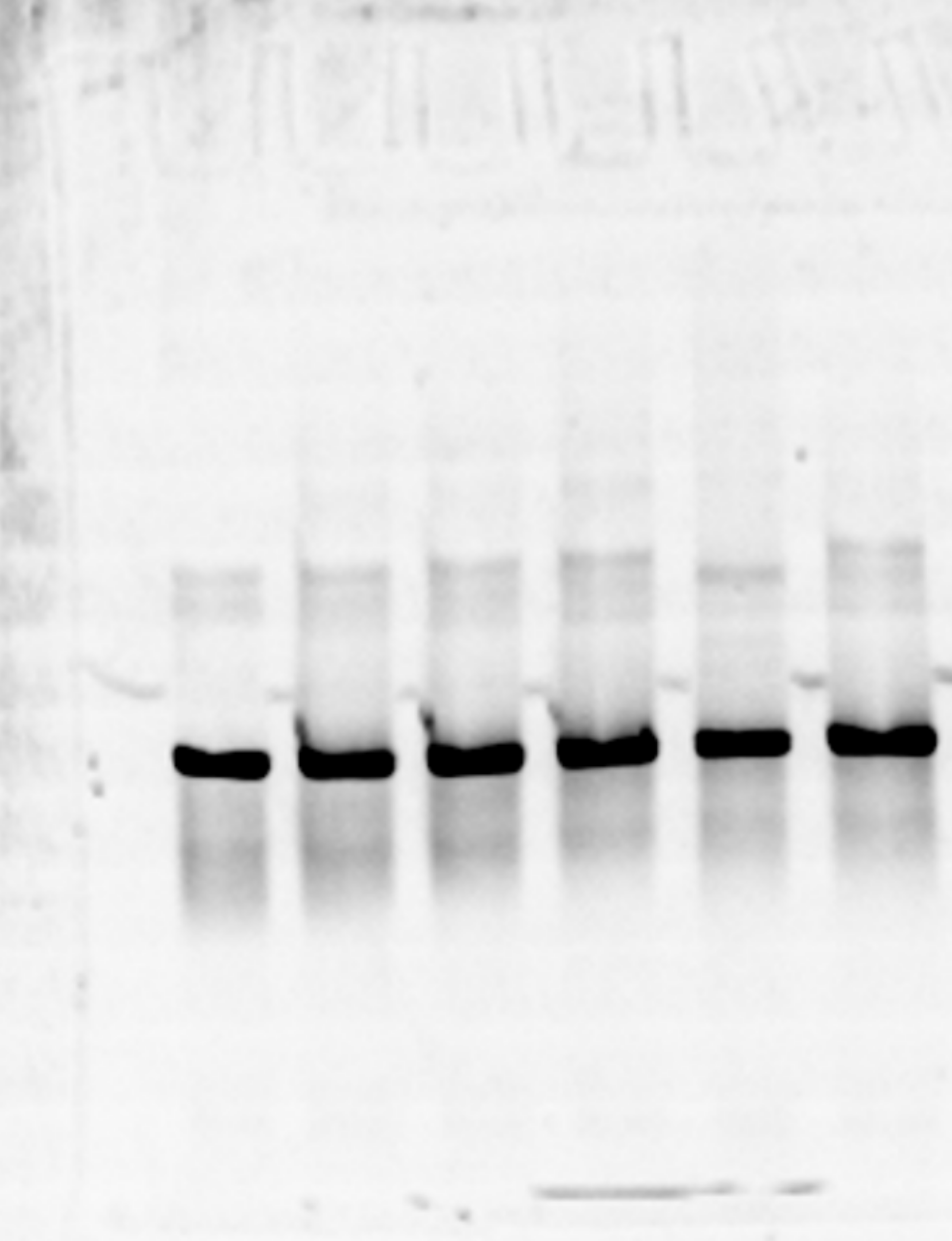

Supplement: Supplementary file 15 — Source data Fig. 6 [file 44320_2025_132_MOESM15_ESM.zip › Figure 6/Figure6B_gel2.png]

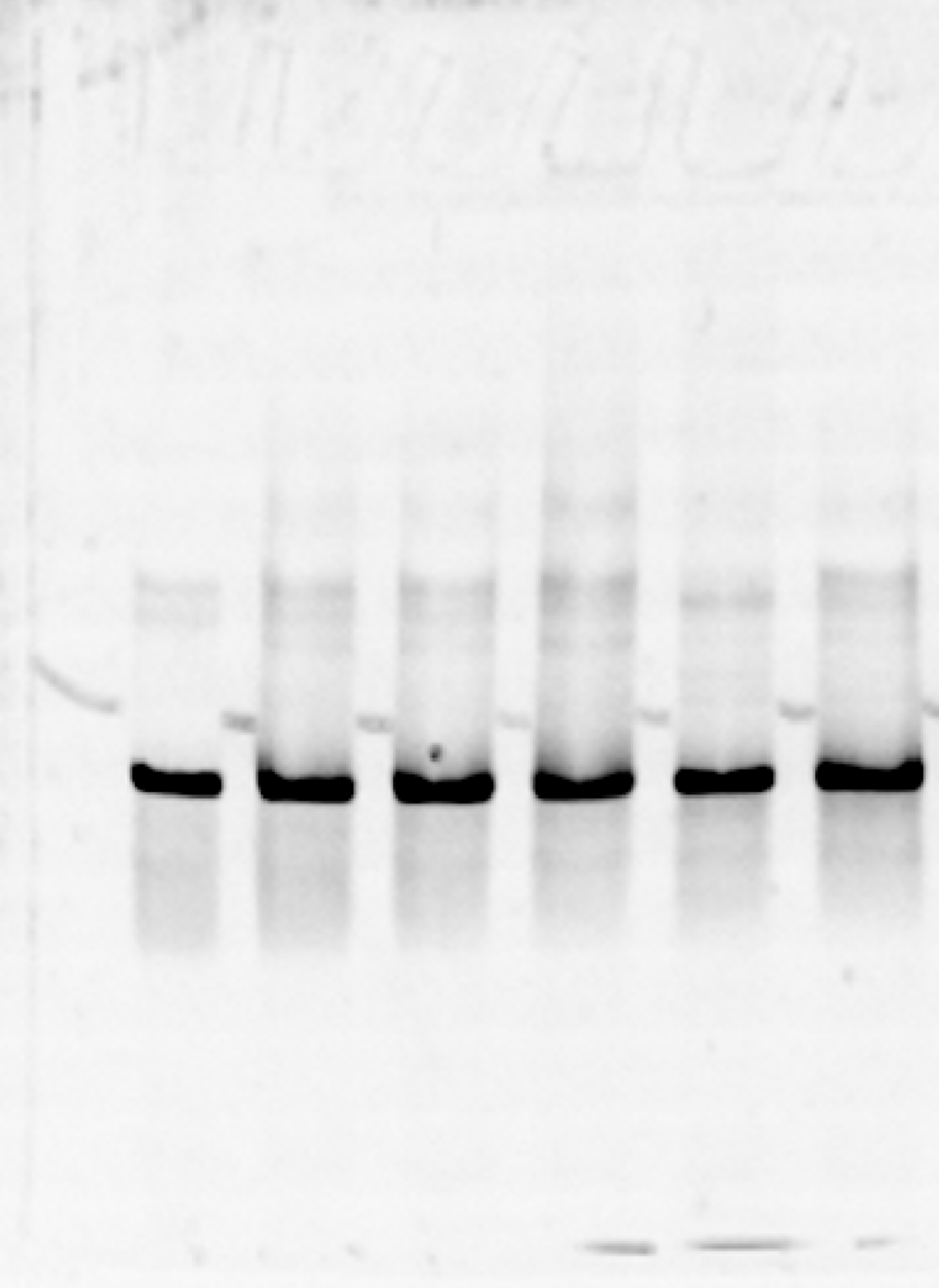

Supplement: Supplementary file 15 — Source data Fig. 6 [file 44320_2025_132_MOESM15_ESM.zip › Figure 6/Figure6B_gel3.png]

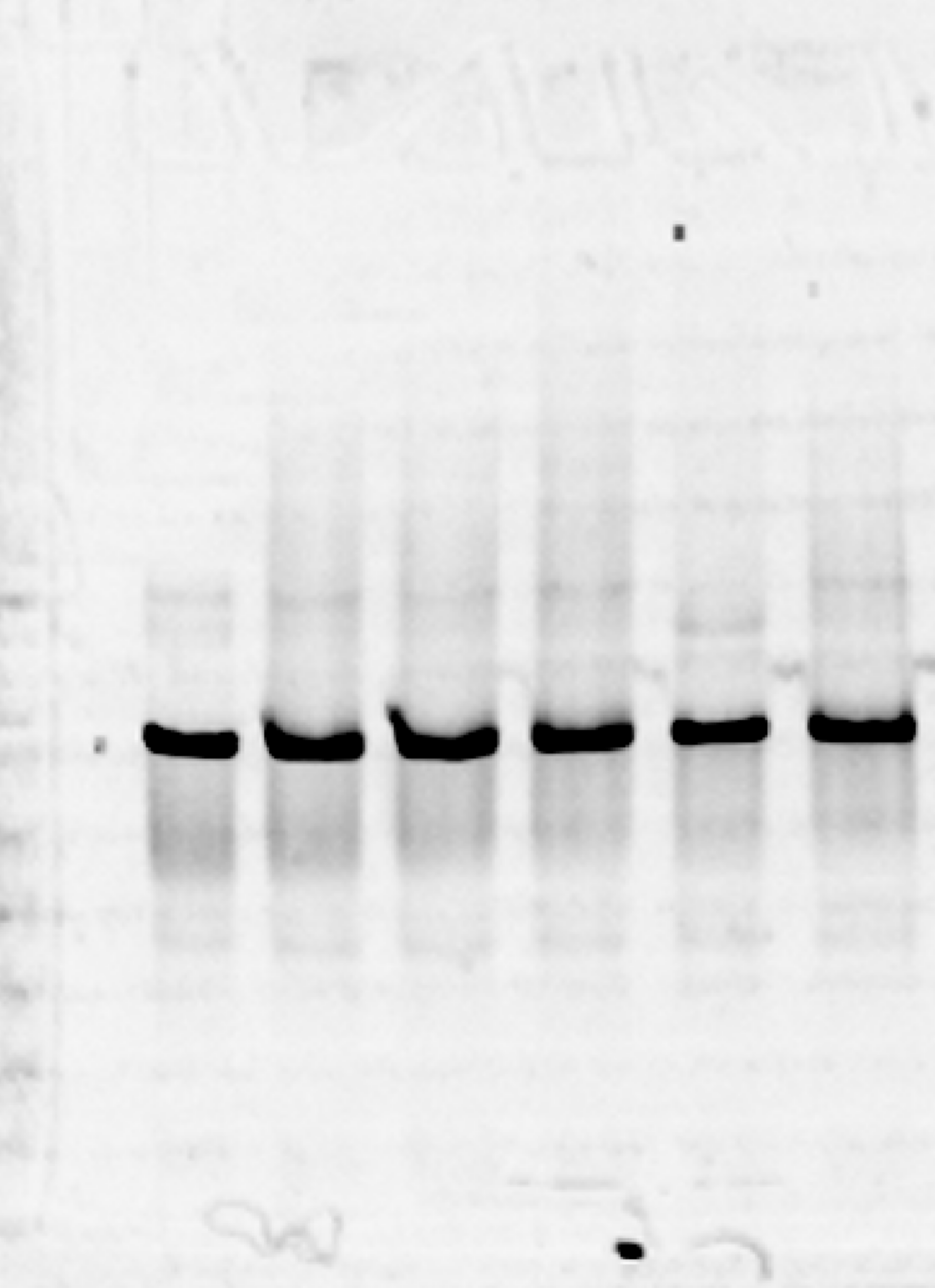

Supplement: Supplementary file 15 — Source data Fig. 6 [file 44320_2025_132_MOESM15_ESM.zip › Figure 6/Figure6B_gel4.png]
