# Supplementary material for: Predicting input signals of transcription factors in Escherichia coli
Source: Mol Syst Biol. 2025 Jul 16;21(10):1371–87. doi: 10.1038/s44320-025-00132-2 (PMC12494820; doi:10.1038/s44320-025-00132-2)
Supplement: Supplementary file 16 — Expanded View Figures [file 44320_2025_132_MOESM16_ESM.pdf]

Expanded View Figures

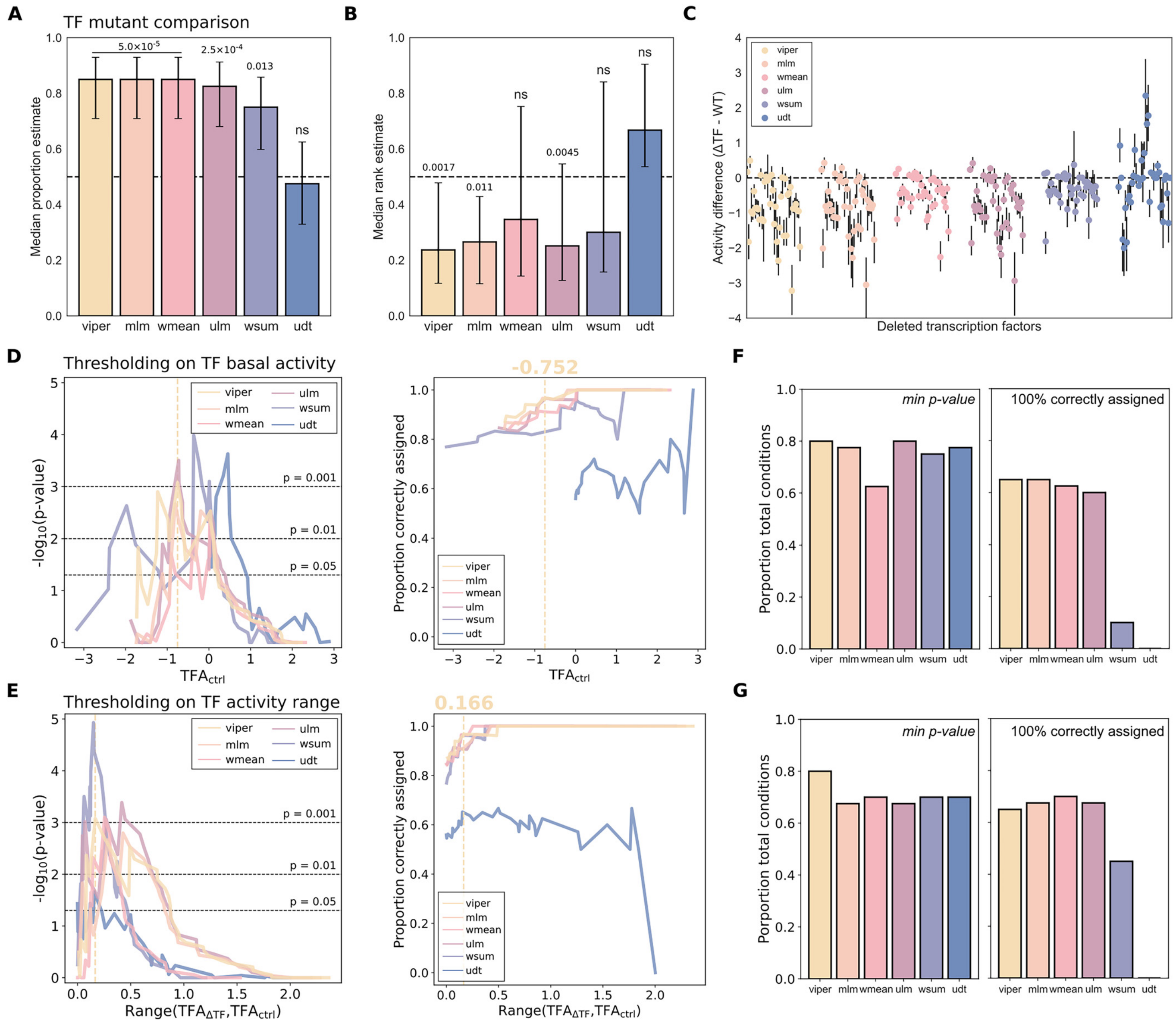

Figure EV1. Validation of transcription factor activity inference methods.

(**A**) Proportion of TF mutant conditions with correct direction inference; i.e., the corresponding TF activity is lower in the TF knockout mutant sample compared to a control ($n = 40$). Bars indicate the 95% Wilson score intervals. (**B**) Median rank estimates of all deleted TFs across all TFs in TF mutant conditions ($n = 40$). Bars indicate interquantile ranges from TF activities across conditions. (**A**, **B**) *ns*: non-significant, Exact *P* values below 0.05 are indicated above the corresponding box from a two-sided binomial test against random distribution (dashed line) with Bonferroni correction. (**C**) TF activity difference (mutant against *corresponding wild-type*) for each TF mutant condition across the six tested methods ($n = 40$). (**D**, **E**) Left: Statistical significance from X^2 test for increased correctly assigned TF activities resulting from dynamical thresholding across ranges of values of (**D**) TF basal activity in the corresponding control condition or (**E**) TF activity range between the control and TF knockout mutant conditions. TFA: Transcription factor activity. Right: Proportion of correctly assigned TF activities from TF mutant conditions across a range of threshold values based on (**D**) TF basal activity in the corresponding control condition or (**E**) TF activity range between the control and TF mutant conditions. Threshold values minimizing the *P* value are shown as dotted vertical lines for the viper method when significant and are written in yellow above the corresponding dotted lines. (**F**, **G**) Proportion of experimental conditions kept after thresholding for (**F**) TF basal activity in the corresponding control condition or (**G**) TF activity range between the control and TF mutant conditions using either the threshold values minimizing the *P* value (left) or the threshold values that allow for 100% of correctly assigned TF activities when possible (right). (**A–G**) The six tested methods are multivariate linear model (mlm), weighted mean (wmean), virtual inference of protein-activity by enriched regulon analysis (viper), univariate linear model (ulm), weighted sum (wsum) and univariate decision tree (udt). All methods are colored following the same color scheme across all panels.

 © The Author(s)

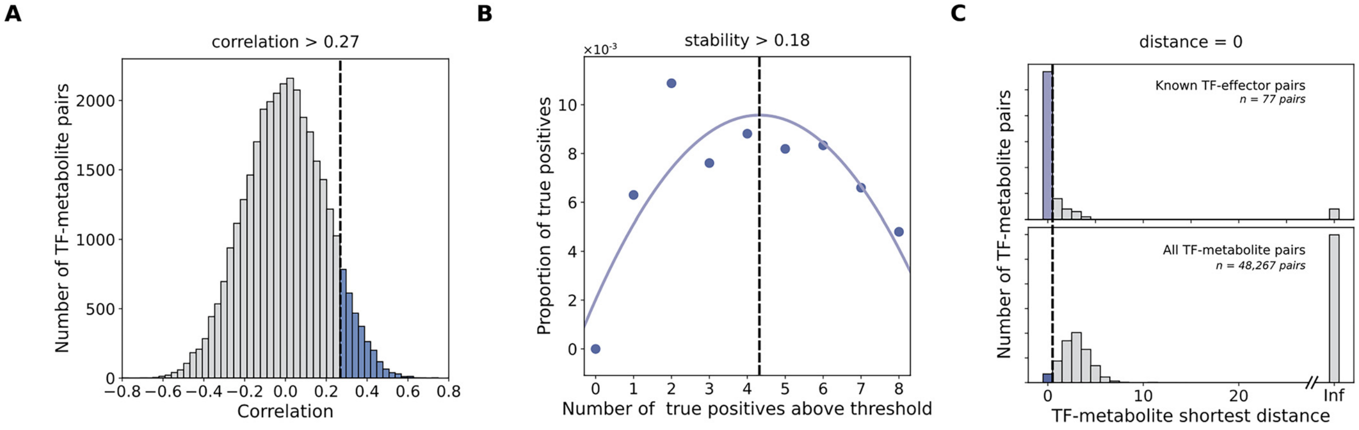

Figure EV2. Filtering approach to identify candidate TF input signals.

(**A**) Distribution of correlation scores across all TF–metabolite pairs. Bins above the False Positive Rate of 0.1 correlation threshold (dotted line) are colored in blue. (**B**) Proportion of known interactions recovered across a range of stability thresholds expressed as number of known interactions above threshold. The stability threshold was determined as the summit of a parabolic fit (dotted line). (**C**) Distribution of TF–metabolite distances for known interactions (top) or all pairs (bottom), with distance threshold of 0 shown as dotted line.

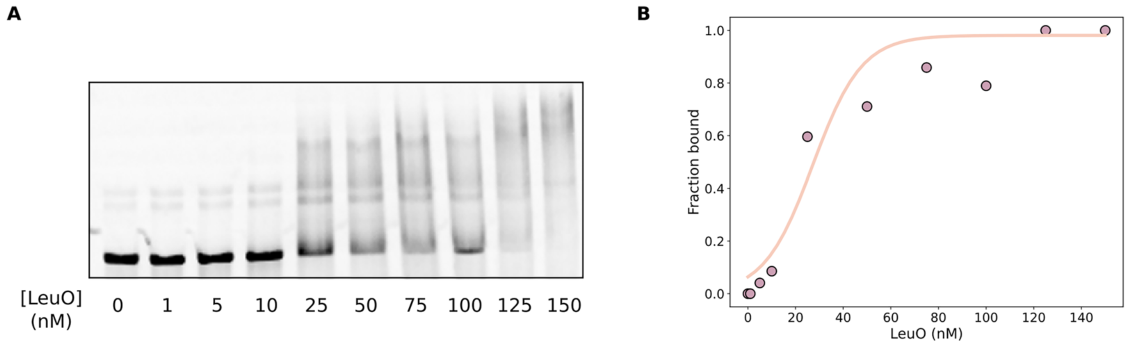

Figure EV3. LeuO binding to the *leuLABCD* promoter region.

(**A**) Gel shift experiment with increasing concentrations of LeuO protein and 0.5 nM of DNA fragment containing the *leuLABCD* promoter region. (**B**) Quantification of fractions of bound DNA from (**A**). The curved line represents a sigmoid fit of the data.

 © The Author(s)

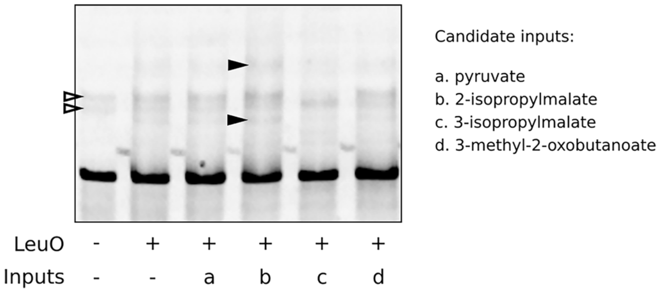

Figure EV4. Gel shift assay to identify LeuO signal molecule.

A representative gel is shown out of four independent gel shift experiments with 0 (−) or 25 nM (+) of LeuO protein and 0.5 nM of DNA fragment containing the *leuLABCD* promoter region. 10 mM candidate inputs were added (+) to test their effect on LeuO binding. Hollow arrows indicate faint bands with unspecific shifts independent of LeuO. Black arrows indicate specific band shifts dependent of LeuO addition.
